# Fabrication of a 3D-Printed Interim Bite Splint for a Hemimandibulectomy Patient: A Case Report

**DOI:** 10.7759/cureus.64120

**Published:** 2024-07-08

**Authors:** Saumil C Sampat, Ishan V Kadam, Ankita Kadam, Kanchan S Sahwal, Sangharsh Ingle

**Affiliations:** 1 Department of Prosthodontics, Dr. G.D. Pol Foundation's Y.M.T. Dental College and Hospital, Navi Mumbai, IND

**Keywords:** 3d-printed prosthesis, masticatory ability, radiotherapy (rt), free vascularised fibular graft, mandibular reconstruction, bite splint, dental malocclusion, mandibular deviation, squamous cell carcinoma (scc), hemimandibulectomy

## Abstract

Mandibular continuity defects can result in varying degrees of cosmetic disfigurement. Restoration of form and function may require surgical reconstruction of the affected area. While surgical reconstruction may improve the overall prognostic outcomes for the patient, the definitive prosthetic phase can commence only after a substantial time lag for adequate hard/soft tissue healing. This interim phase often challenges the patient’s masticatory ability. The traditional reconstruction of hemimandibulectomy defects has its own limitations. This case report describes the fabrication of a 3D-printed bite splint for a patient with limited mouth opening and significant malocclusion due to surgical over-correction. The prosthesis given served as an appliance to improve the masticatory ability of the patient.

## Introduction

Squamous cell carcinoma is the most common pathology affecting the head and neck region including the oral cavity with alcohol and tobacco abuse as commonly implicated etiological factors [1]. Treatment strategies include either surgery, radiation, or a combination of both. Resection in mandibular tumors is dictated by the stage of the disease, a subsite of cancer, the age of the patient, and pre-existing co-morbidities. Radiotherapy in conjunction with such ablative procedures can result in compromised oral function, aesthetics, and comfort with a significant reduction in quality of life. The rehabilitation of such defects should have an interdisciplinary approach integrating the oncosurgeon, reconstructive plastic surgeon, oral surgeon, prosthodontist, speech therapist, nutritionist, etc. to provide customized treatment and prosthetic outcomes. Restoration of near-normal form and function should be the ultimate objective of any treatment rendered. Surgical reconstruction of mandibular continuity defects comes with its own caveats. Mandibular deviation can be caused by fibrosis due to radiotherapy and post-operative soft tissue scar contraction [2]. Significant dysfunction can be encountered in patients with mandibular deviation [3-5]. The case presented here is of a patient who had limited mouth opening and significant dental malocclusion post-surgical reconstruction due to overcorrection of the defect. The prosthesis given served as an interim appliance to improve the chewing ability of the patient.

## Case presentation

**Figure 1:**
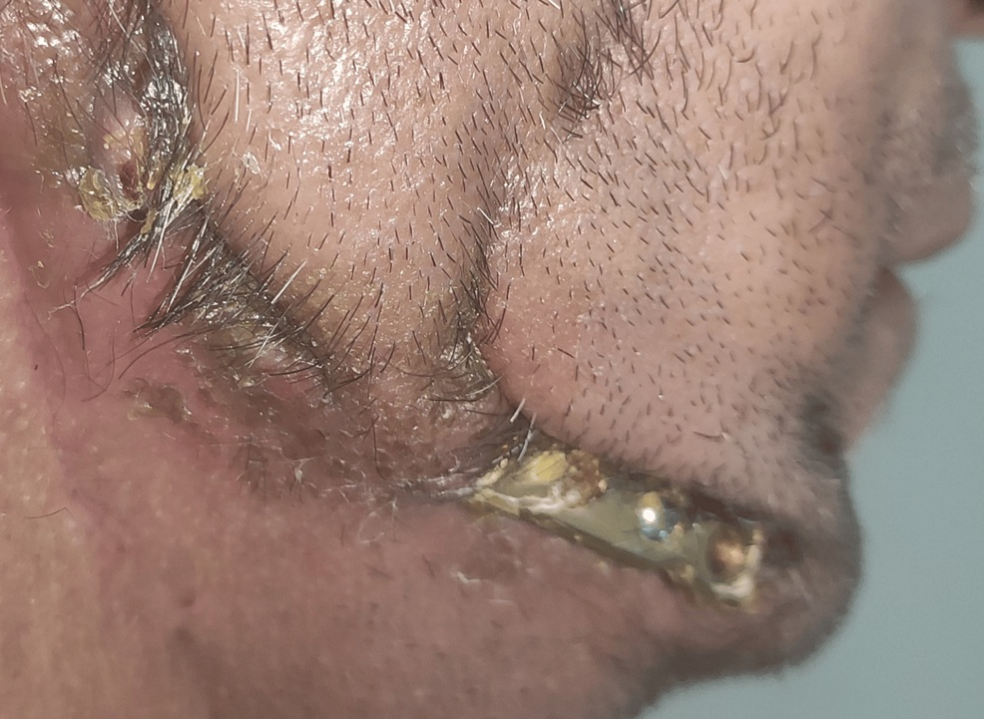
Exposure of reconstruction plate due to infection and tissue contraction post-radiotherapy.

A 41-year-old male with no co-morbidities complained of unresolving ulcerative growth with associated pain on the right posterior lateral border of the tongue for the last three months. Oral examination revealed ulceroproliferative growth over the right lower alveolus extending to the posterior tongue measuring 4 × 1 cm. Biopsy findings revealed well-differentiated squamous cell carcinoma. Computed tomography (CT) scan of the neck with contrast revealed a small well-circumscribed heterogeneously enhancing lesion involving the posterior aspect of the anterior 2/3rd of the root of the tongue on the right side and adjacent floor of the mouth extending posteriorly up to the base, faucial tonsils, retromolar trigone, inferiorly into the oral cavity but not infiltrating the mylohyoid muscle and medially abutting the right genioglossus muscle showing focal loss of fat planes. The imaging features were thus consistent with biopsy findings of neoplastic etiology. The patient underwent right segmental mandibulectomy with modified radical neck dissection (MRND)-III and left free fibula osteocutaneous flap for reconstruction with split-thickness skin graft for defect closure. The patient subsequently reported difficulty chewing due to mandibular deviation and loss of occlusal contacts. Extra-oral examination revealed exposure of the reconstruction plates with cicatrization of the overlying tissues (Figure 1).

**Figure 2:**
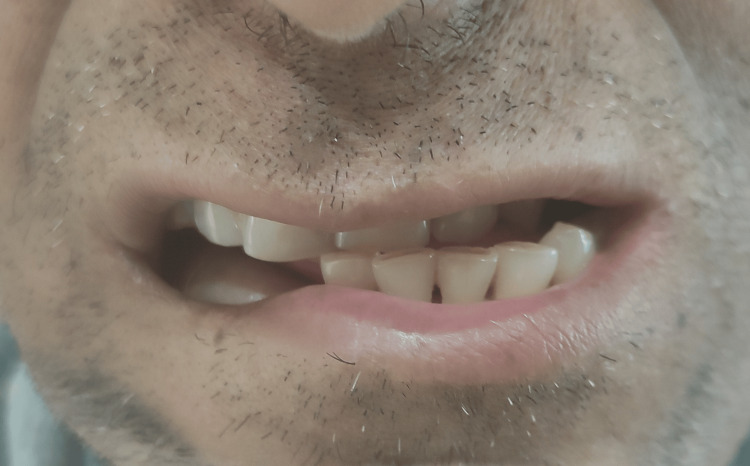
Mismatch of maxillary and mandibular midlines due to overcorrection of resected area.

Intra-oral examination revealed an approximately 1 cm discrepancy in the frontal view between the maxillary and mandibular dental midlines (Figure 2).

**Figure 3:**
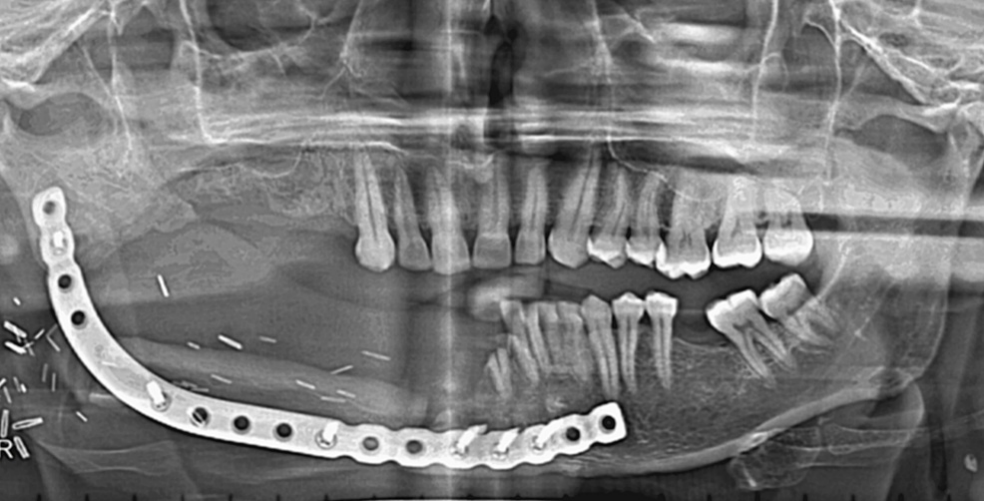
Orthopantomogram (OPG) showing free fibular graft stabilized with a reconstruction plate and screws.

Orthopantomogram evaluation showed partially edentulous maxillary and mandibular arches with a fibular graft stabilized by a reconstruction plate to restore the right mandibular continuity defect (Figure 3).

**Figure 4:**
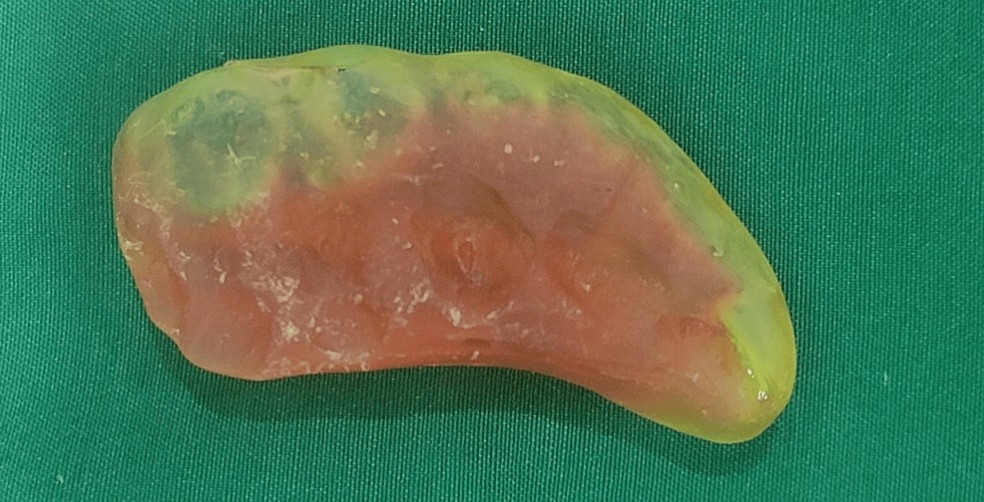
Bite splint as seen from maxillary occlusal view.

Before any prosthodontic intervention could be initiated, the patient was advised to undertake mouth-opening exercises as suggested by Beumer et al. [6]. Due to restricted mouth opening, sectional impressions were made of the 2nd and 3rd quadrants using alginate impression material (Impreceed, GC Corporation, Tokyo, Japan). A bite registration was then made using a putty consistency vinyl polysiloxane impression material (Flexceed, GC India Pvt. Ltd., India) and a triple tray (Capri® Bite Registration Tray, Kerr Corporation, Orange, USA). The models retrieved from the 2nd and 3rd quadrants were scanned along with the bite registration using an extraoral scanner (MEDIT T310, MEDIT Corp., Seoul, South Korea). Meshes of the upper and lower reconstructed models were aligned with the scan of the bite registration using dental computer-aided designing (CAD) software (exocad, exocad GmbH, Darmstadt, Germany). The bite splint was then designed with minimal eccentric interferences on a virtual articulator and fabricated using a 3D printer (NextDent®5100, NextDent B.V., Soesterberg, Netherlands) (Figure 4).

**Figure 5:**
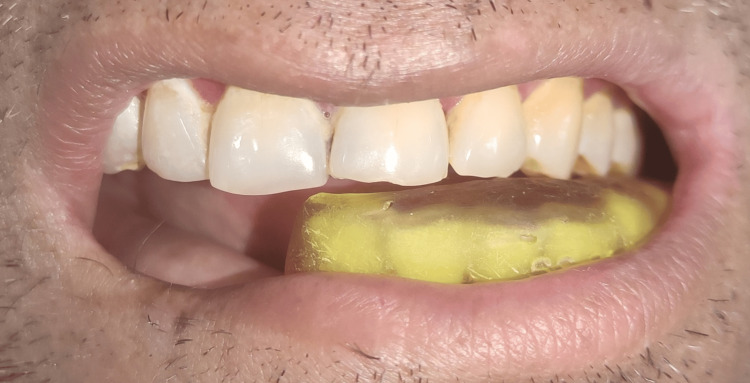
Bite splint in place on mandibular teeth of unresected side, providing stable occlusal platform.

The patient was satisfied with the fit of the splint and was able to chew from the unresected side despite the malocclusion (Figure 5).

## Discussion

Mandibular segmental defects lead to malocclusion, mandibular deviation, TMJ alterations, and soft tissue retraction [7]. The degree of mandibular deviation is dictated by the extent of the lesion, soft tissue involvement, innervation involved, and residual dentition. Prosthetic planning should be an integral part of the overall treatment plan of resection cases to minimize unfavorable prosthetic outcomes. Immediate surgical reconstruction and prosthetic rehabilitation should be the mainstay for such cases. The increased success rate of mandibular reconstruction using plates can be attributed to their improvements in design and manufacturing materials. Traditional reconstruction plates used to stabilize free fibular grafts though a cheaper alternative, suffer from complications such as post-operative plate exposure, screw loosening, infection, and plate fracture [8]. The majority of plate-related complications are due to post-surgery radiotherapy. Ionizing radiation causes occlusion of arteries, causing a decrease in local tissue vascularity with subsequent fibrosis and necrosis of tissues, and infection with subsequent plate exposure [9].

In today's age of digital technology, it is now possible to achieve esthetic and functional treatment outcomes with minimal post-operative complications. Treatment strategies now aim at providing customized results rather than a "one size fits all" approach [10]. Virtual surgical planning (VSP), computer-aided designing (CAD)/computer-aided manufacturing (CAM) cutting guides, stereolithographic models, and navigational surgery can help in visualizing the end result in mind [11,12]. CAD-CAM cutting guides help translate treatments devised by the surgical team into predictable results with precision, accuracy, and reliability. Dynamic navigational surgery can help in positioning osseointegrated implants at specific prosthetic positions, resulting in faster dental rehabilitation [13,14]. Thus, a combination of virtual surgical planning and dynamic navigational surgery can help in the rehabilitation of complex mandibular detects with the best possible outcomes and faster inclusion to normalcy [15]. However, the increased cost, access to technology, and the need for external laboratory support can be a prohibitive factor in the rehabilitation of such cases.

Considering the socio-economic status of the patient in concern, reconstruction was done using titanium reconstruction plates and a free fibular graft. Due to unfavorable reconstructive outcomes causing limited mouth opening, mandibular deviation to the unresected side, infection, and subsequent exposure of the reconstruction plates, masticatory ability was compromised. An attempt was made to provide the patient with a stable interim bite platform using 3D printing as an aid till complete healing occurred.

## Conclusions

While restoration to near-normal aesthetics and function is the end goal of any surgical treatment, limitations due to financial constraints, demographic variations, and access to care can be prohibitive in attaining these goals. Our case report describes a patient who had undergone hemimandibulectomy due to squamous cell carcinoma of the right lower posterior mandibular alveolus and lateral border of the tongue. Due to surgical overcorrection, subsequent infection, and exposure of the reconstruction plate, significant malocclusion was present which hampered the patient's masticatory ability. A 3D-printed interim bite splint was fabricated which helped the patient to chew from the unresected side. Satisfactory masticatory performance was reported by the patient.
